# Antioxidant-Enriched Diet on Oxidative Stress and Inflammation Gene Expression: A Randomized Controlled Trial

**DOI:** 10.3390/genes14010206

**Published:** 2023-01-13

**Authors:** Paola Gualtieri, Marco Marchetti, Giulia Frank, Antonella Smeriglio, Domenico Trombetta, Carmela Colica, Rossella Cianci, Antonino De Lorenzo, Laura Di Renzo

**Affiliations:** 1 Section of Clinical Nutrition and Nutrigenomics, Department of Biomedicine and Prevention, University of Tor Vergata, Via Montpellier 1, 00133 Rome, Italy; 2 School of Specialization in Food Science, University of Rome Tor Vergata, Via Montpellier 1, 00133 Rome, Italy; 3 Ph.D. School of Applied Medical-Surgical Sciences, University of Rome Tor Vergata, Via Montpellier 1, 00133 Rome, Italy; 4 Department of Chemical, Biological, Pharmaceutical and Environmental Science, University of Messina, Viale Ferdinando Stagno d’Alcontres 31, 98166 Messina, Italy; 5 CNR, IBFM UOS, Università Magna Graecia, Viale Europa, 88100 Germaneto, Italy; 6 Department of Translational Medicine and Surgery, Catholic University of the Sacred Heart, Fondazione Policlinico Universitario A. Gemelli, IRCCS, 00168 Rome, Italy

**Keywords:** Mediterranean Diet, nutrigenomics, gene expression, polyphenols, antioxidant, anti-inflammatory activity

## Abstract

The Mediterranean Diet (MedDiet) is associated with beneficial effects against chronic non-communicable diseases (CNCDs). In particular, the content of micronutrients leads to an improvement of the oxidative and inflammatory profiles. A randomized, parallel, controlled study, on 24 subjects, was conducted to evaluate if 2-week supplementation with a mixed apple and bergamot juice (MAB juice), had a positive impact on the body composition, the biochemical profile, and oxidative and inflammatory gene expression (*Superoxide dismutase* (*SOD1*), *Peroxisome Proliferator-Activated Receptor γ* (*PPARγ*), *catalase* (*CAT*), *chemokine C-C motif ligand 5* (*CCL5*), *Nuclear Factor Kappa B Subunit 1* (*NFKB1*), *Vitamin D Receptor* (*VDR*), and *Macrophage Migration Inhibitory Factor* (*MIF*)), respect to a MedDiet. Body composition evaluation analysis showed a gain in lean mass (*p* < 0.01). Moreover, a significant reduction in total cholesterol/HDL index (*p* < 0.01) was pointed out between the two groups. Gene expression analysis highlighted an increase in MIF (*p* ≤ 0.05), PPARγ (*p* < 0.001), SOD1 (*p* ≤ 0.05), and VDR (*p* ≤ 0.05) expressions when comparing MedDiet and MedDiet + MAB juice groups. These data based on the nutrigenomics approach demonstrated that supplementing 2 weeks of MAB juice to the MedDiet could contribute to a reduction in the risk of CNCDs.

## 1. Introduction

The components of the diet, macro and micronutrients, represent factors related to the start of metabolic disorders, such as cardiovascular diseases, type 2 diabetes, metabolic syndrome, and other chronic non-communicable diseases (CNCDs) [1,2].

To counteract CNCD risk factors, it is necessary to adopt a lifestyle medicine, which includes all the actions to be taken before the pathologies become manifest through the adoption of healthy eating habits, possibly integrating foods functional into the diet, and verifying their effects on metabolism and gene expression [3].

The possibility of delaying the onset of CNCDs also lies in the modulation of pathophysiological phenomena dependent on specific micronutrients able to regulate gene expression underlying the phenomenon under observation [4].

Some micronutrients, such as Argine, glutamine, omega-3 fatty acids (n-3), nucleotides, vitamins C and D, and antioxidant compounds, favor the reduction in the inflammation status that characterizes the CNCDs and improves the immune system [5].

The variety of micro and macronutrients, which recombine in a unique way in a food plan adapted to the Mediterranean Diet (MedDiet), contributes to the reduction in CNCDs and cardiovascular mortality, and weight management [6,7]. Indeed, the MedDiet correlates with a healthier body composition and a reduction in waist circumference [8], promoting weight, body mass index (BMI) and fat mass (FM) reduction [9] that also significantly affects the blastocyst euploidy rate [10]. Furthermore, the MedDiet is known to positively influence aging, reduce inflammation, and improve respiratory capacity and endothelial function, thus reducing the morbidity of CNCDs [3].

The high content of antioxidant molecules, fiber, monounsaturated fats (MUFAs), and a suitable ratio of omega-6 (n-6):n-3 fatty acids of typical MedDiet foods contributes to achieving good health [11].

Specific foods and drinks included in the Mediterranean Diet contain natural bioactive compounds with benefits for health and, above all, for the prevention of various chronic pathologies related to inflammation. Olive oil, fish, red wine, fruit, and vegetables contain phenolic compounds and polar lipid bioactives that justify the anti-inflammatory properties of the MedDiet. In particular, vegetables and fruits, foods rich in flavonoids and antioxidants, potentiate the endogenous antioxidant system and show beneficial effects on inflammation and oxidative stress markers [12], such as the pro-inflammatory cytokine Interleukin (IL)-6 [13].

The apple (Pyrus Malus or Malus communis), belonging to the Rosaceae family, contains flavonols in monomeric or oligomeric form, chlorogenic acid, hydroxycinnamic acids, quercetin glycosides, phloretin glycosides and anthocyanins [14].

The most abundant polyphenols in apple flesh are catechin, procyanidin, epicatechin, and phloridzin [15].

Considering the content of essential vitamins (flavonoids, carotenoids, group B vitamins, vitamin C and E, niacin, and folic acid) the apple has antioxidant power, with the possibility of helping to reduce CNCDs [16]. Aprikian et al. showed that by combining the pectins and the phenolic fraction of the apple, plasma and liver cholesterol and triglycerides are reduced. Moreover, cholesterol absorption is better than pectins or phenols utilized alone; the study showed that the consumption of fresh food was beneficial compared to dietary supplements, demonstrating how fruit fibers and phenolic components interact positively [17]. The apple has also been proved to have a central role in the prevention of type 2 diabetes, due to the quercetin present in the peel [18]. According to De Oliviera et al., apple consumption is also known to be associated with weight loss in overweight adult women [19].

Bergamot (Citrus bergamia Risso) belongs to the genus Citrus, to the Rutaceae family [20]. The major components of bergamot are O-glycosylated flavanones, such as eriocitrin, neoeriocitrin, naringin, and neohesperidin; O-glycosylated flavanones esterified with two oxalate molecules, such as neoeriocitrin-di-oxalate, naringin-di-oxalate, neohesperidine-di-oxalate; C-glycosylated flavones and O-glycosylated flavones; HMG-flavanones, especially brutieridin and melitidine [21]. In addition to these compounds, in bergamot, derivatives of caffeic acid and furanocoumarins such as bergaptene and bergamottin are present. In addition to antimicrobial and anti-inflammatory activity by influencing some enzymatic systems, they also mediate antioxidant and diuretic effects, acting as renal phosphatase inhibitors, antispasmodic and vaso-protective, increasing anti-aging and antioxidant gene expression [22]. Furthermore, flavonoids and polyphenolic fractions promote a reduction in glycemia and cholesterol, and determine the mobility of lipids, for greater cardioprotection [23,24].

There is extensive scientific literature reporting the beneficial effects in whole and fresh fruit intake. However, there are few data attesting to the effectiveness of the consumption of fruit juices, and in particular, those composed of little consumed fruits, such as bergamot for its low palatability.

Leopoldini et al., described how brutieridin and melitidine, contained in bergamot, are structural analogs of statins and, for the structure compatible with the active site of the HMG-reductase, block, with a statin-like action, the reduction of coenzyme-A (HMG-CoA) conjugated 3-hydroxy-3-methyl glutaric acid to mevalonic acid [25]. At the same time, an improvement in insulin resistance was also demonstrated as naringenin determines a better absorption of blood glucose by the GLUT4 receptors, increasing the activity of AMP kinase (AMPK) [26].

To our knowledge, there is no study that has evaluated the effects of mixed apple and bergamot juice (MAB juice) on lipid and glycemic profile biomarkers concerning the expression of genes related to antioxidant and anti-inflammatory activity.

Therefore, a randomized controlled dietary intervention study was initiated in healthy human volunteers with the primary aim of verifying the efficacy of 2-week consumption of MAB juice on the modulation of lipid and glycemic profile in blood, and secondarily on the expression of *Macrophage Migration Inhibitory Factor* (*MIF*), *chemokine C-C motif ligand 5* (*CCL5*), *Peroxisome Proliferator-Activated Receptor γ* (*PPARγ*), *Nuclear Factor Kappa B Subunit 1* (*NFKB1*), *Vitamin D Receptor* (*VDR*), *Superoxide dismutase* (*SOD1*), and *catalase* (*CAT*).

## 2. Materials and Methods

### 2.1. Participants and Study Design

The primary aim of the present trial in healthy human volunteers was to assess the effect of MedDiet with or without MAB juice for two weeks on lipid and glycemic blood profiles. The second aim was to examine the effect of MedDiet with or without MAB juice on the inflammasome and oxidative stress, by assessing changes in transcript levels of 7 related genes. Thirty healthy volunteers were consecutively recruited among the population of subjects belonging to the Section of Clinical Nutrition and Nutrigenomics, Department of Biomedicine and Prevention of the University of Rome Tor Vergata, Italy, for a routine medical check-up program [27,28]. The inclusion criteria were as follows: an age between 18 and 65 years, BMI of 19–29.90 kg/m^2^, in good health.

Exclusion criteria included the presence of one or more of the following diseases: acute or chronic metabolic/diabetes/intestinal, cardiovascular/liver/kidney, autoimmune, HIV/AIDS/COVID-19/cancer. Pregnant and lactating women, and anyone taking any type of medication or supplement or following a specific diet were excluded.

Exclusion factors also included frequent or daily consumption of flavonoid-rich beverages such as tea, herbal teas, coffee, cocoa, and fruit juices exceeding 500 mL (as estimated by the food frequency questionnaire).

Finally, twenty-four subjects (sixteen female) completed the study.

Three enrolled subjects did not meet the inclusion criteria: one had a BMI < 19 Kg/m^2^, one had diabetes type 2, and one had a previous history of ischemic heart disease. Three of them decided not to take part in the study again during the first phase.

The sample size was considered adequate to achieve 95% statistical power for detecting a difference in Fold Change value (FC) gene expression of >1.5.

A randomized crossover, controlled study design, with two arms of dietary treatment, was used: arm (1) MedDiet: carbohydrates 55–60% of total Kcal; protein 15–20% of total Kcal of which 50% plant-derived; total fats <30% of total Kcal; saturated fat <10% of total Kcal; polyunsaturated fatty acids (PUFA) 6–10% of total Kcal: 5–6% of total Kcal from n-6 PUFA, and 1–2% of total Kcal from n-3 PUFA; MUFA about 15% of total Kcal; trans-fatty acids <1% of total Kcal; 30 g of fiber; oxygen radical absorbance capacity (ORAC) > 5000 µMolTe; arm (2) MedDiet + 250 mL/day of MAB juice (MedDiet + MAB juice).

**Figure 1:**
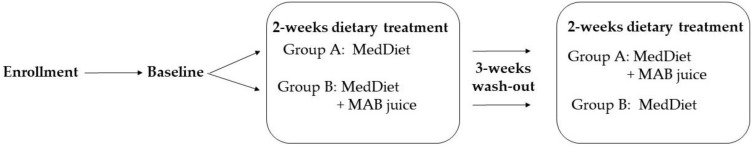
Study design. The randomized crossover study was divided into two treatment interventions: MedDiet, Mediterranean Diet; MedDiet + MAB juice, mixed apple, and bergamot juice. Between the 2-week dietary treatment, a 3-week wash-out period was inserted. Nutritional status was evaluated at baseline. The blood sample for nutrigenomic and biochemical analysis was collected at baseline and the end of each treatment period.

During the study period, enrolled volunteers were randomly assigned to group A following the MedDiet; group B following the MedDiet + MAB juice. After each 2-week dietary intervention, a wash-out of 3 weeks was followed to prevent additive effects on the next treatments. After the wash-out period, the 2-week dietary treatment of the two groups was reversed, as shown in Figure 1.

MAB juice was made by the research team and distributed to enrolled volunteers.

At baseline, an assessment of nutritional status was performed, based on the collection of anthropometric parameters and body composition. Samples for biochemical analysis and genomics evaluation with an analysis of seven genes belonging to oxidative stress and inflammation pathway were collected at baseline and after 2 weeks of MedDiet and MedDiet + MAB juice.

Participants were not blinded to the type of juice they consumed.

Participants were aware of the type of treatment. They were asked not to change their lifestyle and to note any changes during the study. Clinicians assessed any adverse effects and symptoms that were possibly associated with the interventions. No abnormality was presented during the study period.

Informed consent was signed by all participants, by the principles of the Declaration of Helsinki. This trial is registered with ClinicalTrials.gov NCT01890070. The approval of the study was obtained by the Ethics Committee of the Calabria Region Center Area Section (Register Protocol No. 146 17/05/2018). This research received no external funding. All the costs for the entire research protocol and the clinical trial were covered by the own funds of the Clinical Nutrition and Nutrigenomics Section of the Department of Biomedicine and Prevention, deriving from cost savings.

### 2.2. Quality and Quantitative Parameters of Juices

Fresh MAB juice samples consisting of 80:20, *v*/*v* apple and bergamot (flesh and albedo) juice, respectively, were stored in the dark at 4 °C until analyses. Nutritional and functional properties were quantified on three independent samples in triplicate (*n* = 3).

Energy, ash, protein, sodium, salt, fats, humidity, dietary fiber, and carbohydrates were determined according to ISTISAN 1996/34 methods [29].

Total phenols and flavonoids content as well as oxygen radical absorbance capacity (ORAC) assay were carried out according to Smeriglio et al. [30]. Ascorbic acid and polyphenols profiles were characterized by HPLC-DAD and LC-DAD-ESI-MS analysis respectively, according to Smeriglio et al. [30].

### 2.3. Body Composition Measurements

The anthropometric measurements relating to the determination of body weight, height, hip and waist circumferences, and skin folds were carried out by trained personnel following the methods reported in the literature [31]. Bone mineral density (BMD); FM; lean mass (LM); appendicular skeletal muscle mass index (ASMMI) [32]; and resting metabolic rate (RMR) was assessed by Dual-Energy X-ray Absorptiometry (DXA) [33] at baseline. Fat-free mass (FFM); total body water (TBW); extracellular water (ECW); body cell mass (BCM); phase angle (PA) [34]; Sodium (Na); potassium (K); lean mass (LM); metabolic resting rate (BMR); body cell mass index (BCMI) were obtained from Bioelectrical Impedance Analysis (BIA), 101 S (Akern/RJL Systems, Florence, Italy), according to the previously described procedure [35,36], at the beginning and the end of the 2 weeks of MedDiet or MedDiet + MAB juice.

### 2.4. Hematological Sampling and Measurements

After overnight fasting at baseline, and after two weeks of intake of MAB juice, blood was collected for hematological and gene expression analysis. Heparinized venous blood was collected for tests: glycemia, insulin, cholesterol, low-density lipoprotein cholesterol (LDL), high-density lipoprotein cholesterol (HDL), and triglycerides were carried out by the accredited Clinical Chemical Laboratories of the “Tor Vergata” Polyclinic (PTV) of Rome, Italy [37].

Insulin resistance was calculated following applying the homeostasis assessment model of insulin resistance (HOMA-IR):

HOMA-IR = (fasting glucose mg/dL × fasting insulin uU/mL)/405

For transcriptomic analyses, blood was drawn after 12 h of fasting. The blood sample was collected in PAXgene Blood RNA Tubes (PreAnalytiX Qiagen, Hombrechtikon, Switzerland) in the presence of an RNA stabilizer, and stored at −80 °C. Total RNA was extracted using the PAXgene Blood miRNA Kit according to the Total RNA was extracted using the PAXgene Blood miRNA kit according to the manufacturer’s instructions (PreAnalytix Qiagen, Hombrechtikon, Switzerland), and quantified by spectrophotometry (Nanodrop, Wilmington, NC, USA).

### 2.5. Quantitative Real-Time PCR and Data Analysis

RT2 Profiler PCR Arrays were used (Qiagen, The Netherlands) to analyze CAT (GenBank accession No. NM 001752), SOD1 (GenBank accession No. NM 000454), CCL5 (GenBank accession No. NM 002985), PPARγ (GenBank accession No. NM 001145366), NFKB1 (GenBank accession No. NC 000004.12), VDR (GenBank accession No NT 009526.11), MIF (GenBank accession No. NC AY004865). Actin β ACTB (NM_001101) was used as a housekeeping gene. Each qRT-PCR experiment was performed in triplicate and repeated at least twice according to the manufacturer’s instructions (Qiagen, The Netherlands) [38].

### 2.6. Statistical Analysis

Comparisons between groups were made with a paired *t*-test or a nonparametric Wilcoxon, where appropriate. The difference between parameters is expressed as percentages variation (Δ%):

Δ% = [(Z − W)/W] × 100; W = basel value; Z = value after treatment.

For genomic analysis, the comparative threshold (CT) cycle value for each gene was normalized according to the formula ∆CT = CT (gene)−CT (housekeeping gene). The relative gene expression levels were determined using the following formula: ΔΔCT = ΔCT sample −ΔCT calibrator. Expression fold change (FC) = 2^−ΔΔCT^ was used. Gene expression was considered significant respect baseline, when the absolute FC value was of at least ±2.0, and *p*-value ≤ 0.05.

The statistical package SPSS 22.0 software Inc. (Chicago, IL, USA) was used for the analysis and a *p*-value < 0.05 was considered significant.

## 3. Results

### 3.1. Nutritional and Functional Properties of MAB Juice

Tables S1 and S2 show the results concerning the nutritional and functional features of the MAB juice. Being a juice, it is made up of 91.03% water and has a reduced ash and protein content, and very low salt (0.002%). Fats are practically absent, and carbohydrates consist exclusively of sugars (8.55%). Indeed, the fiber content, being a first-pressing fruit juice, is very low (<0.5%). These nutritional characteristics give the product an excellent energy profile having a low-calorie content (35 kcal/100 g).

The MAB juice was also characterized from a functional point of view by determining the total phenols and flavonoid content and the ORAC value, i.e., the scavenging capacity of the MAB juice against free oxygen radicals, a very important antioxidant test to determine a food antioxidant capacitance. Furthermore, the content of Vitamin C, well known for its marked antioxidant effects, and the polyphenolic profile of the juice were determined to evaluate qualitatively and quantitatively which were the most representative polyphenols of the MAB juice, to evaluate what could be the contribution of the most abundant compounds of this particular mix, consisting of 80% apple juice and 20% bergamot juice, to the health effects found in the present study.

As can be seen from Table S2, the MAB juice shows a high content of Vitamin C (422.02 mg/L), total phenols, and flavonoids (1263.16 mg GAE/L and 385.38 mg QE/L), which may explain the high ORAC value found (5964.29 µmol TE/L). The LC-DAD-ESI-MS analysis of the polyphenolic profile upheld these preliminary results, evincing that total polyphenols (484.87 mg/L) were widely contained in the MAB juice, with the following most abundant compounds: chlorogenic acid, procyanidin B2, epicatechin and 4-p-coumaroylquinic acid, characteristic of the apple juice, and neoeriocitrin, naringin, neohesperidin, meltidin, naringin-di-oxalate, brutieridin and neohesperidin-di-oxalate, characteristic of the bergamot juice (compounds reported in bold in Table S2). In this regard, it is interesting to observe how, despite the preponderance of the apple juice (80% vs. 20% of bergamot juice) in the MAB juice tested in the present study, the predominant compounds, in quantitative terms, belong to the bergamot juice, especially in order of abundance: brutieridin, meltidin, and naringin. Among other things, it should be emphasized that this profile can be attributed solely to this particular mix containing bergamot juice obtained by squeezing, at the same time, fruit flesh and albedo.

### 3.2. Clinical Trial

Of 30 enrolled participants, 24 subjects (16 female, and 8 male) were eligible for the study. Table 1 reports the baseline body composition evaluation of the 24 participants.

From DXA data, the body fat mass percentage was 27.86% (±6.91), while the total percentage of obese and pre-obese, according to the classification of fat mass [39] was 36.36%.

Table 2 shows the average distribution of macronutrients in the two diets.

The diet in both periods followed the reference values, except for the dietary fiber intake, which was lower than the recommendations. No differences were observed between pre-study and during-study intakes, except simple carbohydrates, as expected for additional consumption of MAB juice. However, the amount of simple carbohydrates did not reach the limit of 10% of daily calories.

There were no changes in the study outcomes after the start of the trial.

Table 3 shows the comparison between the nutritional status assessment values, related to the anthropometric parameters and the body composition, in the two groups. No significant changes were observed compared with the baseline.

The results of the body composition evaluation by BIA analysis showed significantly increased lean mass values (*p* < 0.01).

A comparison of biochemical analysis between MedDiet and MedDiet + MAB juice groups is reported in Table 4. No meaningful changes were noted compared to the baseline.

A significant reduction in total cholesterol/HDL index (*p* < 0.01) between the two groups was observed. No meaningful difference was noted in the other evaluated parameters.

**Figure 2:**
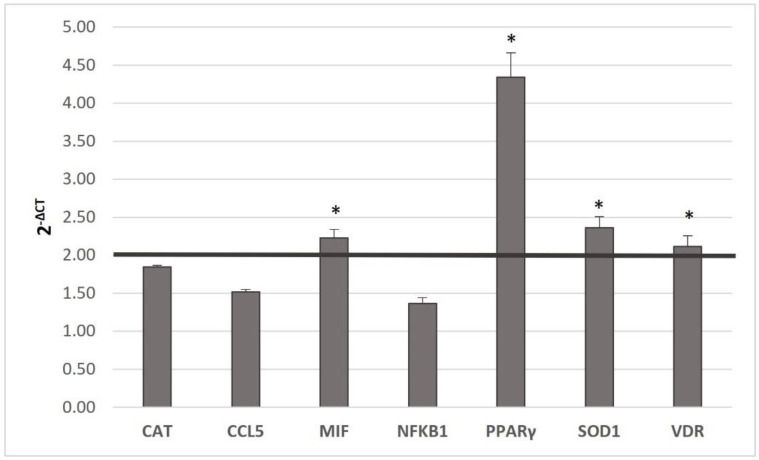
Gene expression between MedDiet and MedDiet + MAB juice. Gene expression is expressed as 2^−ΔCT^. A paired *t*-test was made to assess differences. Statistical significance was assigned as * *p* < 0.05. Abbreviations: *Catalase* (*CAT*); *Chemokine C-C motif ligand 5* (*CCL5); Macrophage Migration Inhibitory Factor* (*MIF*); *Nuclear Factor Kappa B Subunit 1* (*NFKB1*); *Peroxisome Proliferator Activated Receptor γ* (*PPARγ*); *Superoxide Dismutase* (*SOD1*); *Vitamin D Receptor* (*VDR*).

Seven Gene expression changes of the seven genes concerning the assumption of MedDiet and MedDiet + MAB juice were analyzed (Figure 2).

*MIF* (*p* ≤ 0.05), *PPARγ* (*p* < 0.001), *SOD1* (*p* ≤ 0.05), and *VDR* (*p* ≤ 0.05) expressions in-creased significantly (*p* ≤ 0.05) in the comparison between MedDiet, and MedDiet + MAB juice. No significant *CAT*, *CCL5*, and *NFKB1* variations were observed in both conditions.

## 4. Discussion

To date, the MedDiet is the only well-studied dietary pattern. It is known that genetic predisposition may influence stroke incidence, several risk factors such as lipid profile and glycemia, but also emerging ones such as telomere length and emotional eating behavior. MedDiet Adherence could have positive effects on these risk factors [40].

Furthermore, glycemia values in patients with type 2 diabetes mellitus are better if the MedDiet is combined with genetic data to obtain the personalized composition of the macronutrients of the diet [41]. The implementation of a MedDiet may result in the preventive treatment of degenerative diseases and an improvement in life span, a net gain in health, and a reduction in total lifetime costs [42]. The MedDiet is associated with a reduction in all-cause morbidities and mortality. Vegetables, legumes, cereals, fish, fruits, and nuts are the main components of the MedDiet that can help to preserve good health, connecting nutrient metabolism, gut microbiota, and the immune system [43].

Despite the fact that whole fruit assumption has been related to various health effects, few studies are currently available comparing the benefits associated with their consumption and their derivatives, such as juices. One of the advantages of the juice is certainly its ease of consumption. Its moderate intake could make it a very interesting option to benefit from the healthful effects of the fruit. This assumes an even greater value in cases in which fruits are not normally consumed, allowing these subjects to almost satisfy the daily recommendations completely [44].

A study based on data from the 2013–2016 National Health and Nutrition Examination Survey reported that one hundred percent of fruit juice consumers had a ten percent higher Health Score Food Index [45] with respect to non-consumers, and an increased intake of several nutrients (vitamins D and C) and minerals (potassium, calcium, and magnesium).

This has led to our study on volunteers with homogeneous lifestyles and habits and in a balanced food context who were subjected to the intake of the MedDiet for two weeks with or without the supplementation of MAB juice.

Although there are a lot of epidemiological, in vitro, and animal studies on the health effects of apple juice, clinical studies have only recently been carried out to verify the transferability of these results to humans. In particular, it has been shown that apple polyphenols, thanks to their strong antioxidant properties, prevent damage to lung tissue induced by smoking and can reduce LDL oxidation [46,47]. By acting on the stress oxidative reduction, they can decrease the aging-induced cellular damage and perform a preventive activity in several CNCDs [48]. These health effects are mainly attributable to five classes of polyphenols: flava- and flavonols, anthocyanins, phenolic acids, and dihydrochalcones [49,50]. Their concentrations change greatly according to the part of the fruit taken into consideration as well as according to the fruit cultivar, harvest, and storage [49]. Despite the possible depletion in polyphenols, recent studies have shown that the fruit juice nutritional value is similar to that of the whole fruit, except for the fiber and vitamin C content [51]. Wruss et al. demonstrated that the consumption of unfiltered apple juice increases the blood and urine concentrations of phenolic compounds, with elevated interindividual variability [52].

We measured body composition by BIA under controlled and standardized conditions with study participants randomly allocated to the MedDiet or MedDiet + MAB juice.

No variation was shown between BMI and waist circumference after MedDiet + MAB juice concerning MedDiet, according to Barth et al. [53]. A significant increase in lean mass was observed comparing MedDiet vs. MedDiet + MAB juice (*p* < 0.01). The mechanisms of how fruit juice modulates body lean mass might include the regulation of lipid and glycemia metabolism.

Few studies are currently available that have examined different parameters of glucose homeostasis using single doses of apple juice. Ravn-Haren et al. [54] highlighted the effects of apple juice on several cardiovascular disease risk factors. The authors did not report any statistically significant effect of such supplementation on insulin, insulin-like growth factor, and insulin-like growth factor-binding protein 3 concentrations or triglyceride levels [54].

In the present study, no significant changes were shown between MedDiet and MedDiet + MAB juice, regarding glycemia (MedDiet vs. MedDiet + MAB juice Δ% = 0.94, *p* = 0.73) and insulin (MedDiet vs. MedDiet + MAB juice Δ% = 9.30, *p* = 0.36). According to White et al. [55] who used 100% apple juice, our data showed no variation in glycemia.

Our results also agree with data observed by Viera et al. [56], where the consumption of 300 mL was not associated with an increase in plasma glucose levels.

Furthermore, our results on HOMA-IR (MedDiet vs. MedDiet + MAB juice Δ% = 11.11, *p* = 0.30) confirmed the observation on bergamot effects. It has been demonstrated that one of the bergamot flavonoids, naringin, improves insulin sensitivity and glucose tolerance [57].

Hyson et al. [58], demonstrated that administering 375 mL of apple juice, with no added sugar, per day for six weeks to healthy volunteers did not induce any statistically significant variation of total cholesterol, LDL-C, HDL-C, apolipoprotein AI or B levels, although this intervention significantly reduced the susceptibility of LDL to oxidation and the overall production of peroxidized lipids. Similar results were also reported by Barth et al. [53] and Soriano-Maldonado et al. [59] in studies with a similar design on the lipid parameters.

Given the results of the present study in terms of the statistically significant reduction in ColTOT/HDL (MedDiet vs. MedDiet + MAB juice Δ% = −20.91, *p* < 0.01), a key role of bergamot juice could be hypothesized. Miceli et al. showed that chronic administration of bergamot juice was effective in preventing diet-induced hyperlipidemia in rats, decreasing serum cholesterol, triglycerides, and LDL, and increasing HDL levels. Furthermore, histopathological observations showed the ability of bergamot juice to counteract liver damage, suggesting a promising use of this juice in cardiovascular disease prevention [23]. This promising activity could be attributable to brutieridin and melitidin, two of the most abundant flavonoids detected in MAB juice, which were described by Leopoldini et al. [25] as structural analogs of statins, and to naringin and hesperedin, which can reduce the enzymatic activity of the Acyl-CoA cholesterol acyltransferase, inhibiting the lipoproteins assembly [60].

The MedDiet is a diet with an elevated antioxidant power, and positively modulates antioxidant and anti-inflammatory genes [61]. The MedDiet components affect transcription factors, which could have an effect on specific gene pathways [62].

However, few studies have evaluated how apple juice consumption influences the inflammation markers [53,54]. Bart et al. [53] found a relationship between apple juice consumption and IL-6-174 G/C polymorphism, that led to a change in body fat percentage. Soriano-Maldonado et al. [59], observed a significant difference in intercellular adhesion molecule 1 and cell adhesion protein vascular 1, after a daily consumption of an enriched vitamin C and polyphenols apple juice for four weeks [59].

Ravn-Haren et al. [54] highlighted a decrement in the glutathione peroxidase activity after apple juice consumption [54].

Jung et al. [63] analyzed the in vitro effect of apple juice on human gene expression, observing a significant inhibition of the expression of NF-jB-regulated pro-inflammatory genes (*CXCL9*, *CXCL10*, *TNFα*, *IL-1b*), inflammation-relevant enzymes (*CYP3A4*, *COX*-2) and transcription factors (*IRF1*, *STAT1*).

The SIRT1 activation could be responsible for the flavonoid anti-inflammatory activity contained in the bergamot juice. Furthermore, the increase in mRNA transcripts resulting from the LPS-induced inhibitory effects, and also a modulation of some genes, such as *IL-8*, were observed [64,65].

Risitano et al. [66] reported that the bergamot juice flavonoid fraction blocks the *IL-6*, *IL-1b* and *TNFα* gene expression and secretion. Moreover, it reverses the p65 acetylation in LPS-stimulated THP-1 cells.

These anti-inflammatory effects were corroborated by animal studies, which showed that a daily supplementation of bergamot juice extract in a mice experimental colitis model significantly decreased the IL-1β, NK-kbp65, p-JNK, TNF-α colon levels, and the nitrotyrosine positive staining degree [67].

Inflammation regulates many gene pathways. In particular, *CCL5*, often called RANTES, is a chemokine involved in the pathology of inflammatory disease [68].

In our study, no significant *CAT*, *CCL5*, and *NFKB1* expressions were observed in MedDiet vs. MedDiet + MAB juice.

In our results, it was shown that *SOD1* expression significantly increased (*p* ≤ 0.05), between MedDiet, and MedDiet + MAB juice. *SOD1* and *CAT* activate the endogenous antioxidant defense system, due to the intake of phenolic-rich food [69].

A significant variation in *MIF* (*p* ≤ 0.05), a gene related to inflammation and immune-system activity, was observed [70]. It acts by suppressing the anti-inflammatory effects of glucocorticoids.

*MIF* induces NF-kB via activation by the trigger to the mitogen-activated protein kinase (MAPK) and phosphoinositide 3-kinase signaling pathways [71]. *NFKB1*, a pleiotropic transcription regulator, is activated by cytokines, free radicals, and viral or bacterial products. It is also involved in several processes such as immunity, inflammation, cell growth, and apoptosis [72]. Furthermore, this lymphokine forms in the cytosol together with the protein JAB1 a complex near the peripheral plasma membrane that could highlight a role in integrin pathways [70]. Glucocorticoids reduce inflammatory and immune responses but also act on carbohydrate metabolism, as well as on lipids and proteins, increasing catabolism. Therefore, the greater expression of *MIF*, due to the intake of MedDiet + MAB juice could have a favorable action on the body, reducing the catabolic effects determined by glucocorticoids released in a stressful situation.

We highlighted that *PPARγ* expressions increased significantly (*p* < 0.001) in the comparison between MedDiet, and MedDiet + MAB juice. *PPARγ* is involved in several diseases, such as diabetes, atherosclerosis, obesity, and cancer [73]. *PPARγ* is correlated with the maturation and function of lymphocytes, monocytes/macrophages, and dendritic cells, and it may be a key regulator of foam cell gene expression. Moreover, inhibition of *PPARγ* attenuates the proinflammatory cytokines, and its activity can be modulated by a variety of natural compounds [74].

An increase in *VDR* levels (*p* ≤ 0.05) was observed when comparing MedDiet and MedDiet + MAB juice. The *VDR* gene encodes the nuclear hormone receptor for vitamin D3, which is a member of the superfamily involved in mineral metabolism [75], and in energy metabolism, muscle hypertrophy, immune response, and cancer [76].

The protein encoded by this gene also functions as a receptor for lithocholic acid, a particularly important bile acid since it is produced in greater quantities during the oxidative processes of cholesterol and whose anti-aging and antitumor function has recently been discovered [77]. Moreover, *VDR* exerts renoprotective effects mediated by vitamin D, including antiproteinuric, antifibrosis, anti-inflammatory effects [78], and prevents podocyte damage [79].

It is also known that glucocorticoids reduce the *VDR* expression. An increase in this gene expression would confirm the high expression of the *MIF* gene which determines an attenuation of the effect of glucocorticoids.

The results of the nutrigenomics analysis on some genes of inflammation and oxidative stress help to understand more clearly the picture of the lipid and glucose profile.

The Pearson coefficient reported a positive correlation between *SOD1* and *PPARγ* (R = 0.89, *p* < 0.001) and *SOD1* and *VDR* (R = 0.91, *p* < 0.001) expression, respectively, in the MedDiet + MAB juice group.

Since in healthy volunteers, the repair mechanisms of oxidative processes were physiological and efficient, making it difficult to evaluate small variations, it will be necessary to extend the intake time of 250 mL per day of MAB juice to at least one month, in addition to a MedDiet. Our data should be confirmed on a larger number of subjects, with a prospective long-term trial.

## 5. Conclusions

Overall, the results obtained and the literature data currently available show that the consumption of MAB juice, inducing a response on different markers, could positively affect several targets related to the risk of developing CNCDs.

For some antioxidant and anti-inflammatory markers, probably the two juices act synergistically, thanks also to the very different polyphenol profiles that they have, but for others such as those involved in lipid-lowering effects, apple juice appears to have very modest effects, hence the importance of balancing it with bergamot juice, which instead seems to play a pivotal role.

Our study results aligned with those reported by Papandreou et al. [80], on glycemia levels and HOMA-IR. In the case of orange juice, with a different form, it does not affect the postprandial glycemia and insulin in non-diabetic young women. Moreover, based on a systematic review and a meta-analysis of prospective cohort studies, conducted by Tsilas, et al. [81] the fructose-containing sugars, regardless of dietary form, are not definitely associated with an increased type 2 diabetes mellitus risk.

A limitation of this study is represented by the small sample. Therefore, proteomics experiments, analysis of the related signaling pathways, and serum/plasma markers could be investigated. Finally, among the future prospects, the effects of MAB juice on pathological populations will also have to be investigated.

Our findings support, for the first time based on a nutrigenomics prospective, that the supplementation of MAB juice to a MedDiet could contribute to the reduction in the risk of CNCDs if taken regularly. Even if clinical trials to validate the relationship between whole fruits or juice (oranges, apples, and berries) and prevention of type 2 diabetes mellitus risk should be investigated in the future, our findings on the combination between MedDiet and MAB juice, and recent scientific evidence propose that the fruit juice assumption is not harmful to health. The high antioxidant and phytochemical levels and fiber content of fruit juice could be possible reasons for the neutral effects on glycemia and insulin levels. Further research is necessary to assess this issue.

## Figures and Tables

**Table 1:** Baseline body composition evaluation of the participants at the beginning of the study.

Parameters	Mean ± DS (Min–Max) *n* = 24 (F = 16)
Age	30.00 ± 5.24 (23.00–41.00)
BMD (g/cm^2^)	1.18 ± 0.13 (1.02–1.43)
FM (%)	27.33 ± 6.84 (17.70–39.00)
FM (Kg)	15.24 ± 6.47 (1.60–29.05)
LM (Kg)	41.60 ± 9.80 (30.99–59.30)
ASMMI	6.26 ± 1.67 (2.58–8.54)
RMR by DXA (Kcal)	1466.47 ± 237.13 (1148.38–1884.99)

Results are expressed as mean ± standard deviation and minimum (min) and maximum (max) for each parameter. Abbreviations: standard deviation (SD); bone mineral density (BMD); fat mass (FM); lean mass (LM); appendicular skeletal muscle mass index (ASMMI); resting meta-bolic rate (RMR).

**Table 2:** Comparison between macronutrient intake and total antioxidant capacity of MedDiet and MedDiet + MAB juice.

Macronutrients	MedDiet Mean ± SD *n* = 24 (F = 16)	MedDiet + MAB Juice Mean ± SD *n* = 24 (F = 16)	*p*	Reference Values
Calories	1720.27 ± 632.99	1807.77 ± 632.99	0.48	-
Proteins (g)	76.16 ± 28.93	76.63 ± 28.93	0.92	-
Proteins (% of calories)	18.21 ± 4.49	17.33 ± 4.10	0.44	15–20% Kcal/die
Carbohydrates (g)	247.75 ± 90.09	269.12 ± 90.09	0.22	-
Carbohydrates (% calories)	54.63 ± 11.01	60.52 ± 11.04	0.01 *	45–60% Kcal/die
Fat (g)	52.93 ± 31.58	53.13 ± 31.58	0.97	-
Fat (% of calories)	26.25 ± 9.35	25.17 ± 9.01	0.52	20–35% Kcal/die
Saturated fat (% of energy)	7.09 ± 7.73	6.73 ± 3.59	0.74	<10% Kcal/die
Fibers (g)	22.34 ± 8.91	23.54 ± 8.91	0.46	>25 g/die
ORAC (µMolTe)	8891.51 ± 6984.96	10,382.01 ± 6984.96	0.35	5000/die

Results are expressed as mean ± standard. Reference values were based on Dietary Refer-ence Intakes (DRI). A paired *t*-test was made to assess differences. Statistical significance was assigned as * *p* < 0.05. Abbreviations: Mediterranean Diet (MedDiet); mixed apple and bergamot juice (MAB juice); Standard Deviation (SD); Oxygen radical absorbance capacity (ORAC).

**Table 3:** Comparison of the nutritional status assessment values between MedDiet and Med-Diet + MAB juice groups.

Parameters	Baseline *n* = 24 (F = 16)	MedDiet Mean ± SD *n* = 24 (F = 16)	MedDiet + MAB Juice Mean ± SD *n* = 24 (F = 16)	*p*
Weight (kg) ^b^	65.40 ± 13.90	60.74 ± 10.62	60.21 ± 10.63	0.11
BMI (kg/m^2^) ^b^	23,17 ± 2.61	21.78 ± 2.23	21.84 ± 2.25	0.11
Waist circumference (cm) ^a^	77.35 ± 12.23	69.98 ± 6.11	70.02 ± 7.18	0.96
Hip circumference (cm) ^a^	95.80 ± 4.91	94.18 ± 5.35	94.41 ± 4.31	0.74
Waist-to-hip ratio ^b^	0.80 ± 0.09	0.74 ± 0.05	0.74 ± 0.06	0.93
Resistance (Ohm) ^a^	559.50 ± 110.82	577.73 ± 45.20	568.45 ± 68.92	0.48
Reactance (Ohm) ^b^	63.17 ± 11.65	58.78 ± 8.13	58.18 ± 8.57	0.40
FFM (kg) ^b^	51.10 ± 13.53	47.87 ± 8.87	47.13 ± 9.33	0.33
TBW (L) ^b^	37.40 ± 9.90	34.96 ± 6.58	34.48 ± 6.85	0.15
ECW (L) ^a^	16.33 ± 4.23	15.86 ± 2.86	16.07 ± 3.16	0.39
BCM (kg) ^b^	28.82 ± 8.14	25.45 ± 5.49	24.35 ± 5.64	0.93
FM (kg) ^a^	14.30 ± 2.65	13.03 ± 3.79	13.01 ± 3.19	0.97
PA ^a^	6.48 ± 0.68	5.81 ± 0.70	5.84 ± 0.69	0.83
Na/K ^a^	0.97 ± 0.10	1.09 ± 0.13	1.10 ± 0.10	0.76
LM (kg) ^b^	35.03 ± 9.81	26.35 ± 5.87	30.67 ± 6.65	<0.01 *
BMR (kcal) ^b^	1581.23 ± 235.56	1488.23 ± 159.25	1471.28 ± 160.01	0.72
BCMI (kg/m^2^) ^a^	10.08 ± 1.89	8.85 ± 1.02	9.01 ± 1.33	0.44
Biceps fold ^b^	6.65 ± 4.27	6.48 ± 4.15	4.97 ± 1.97	0.11
Triceps fold ^a^	11.77 ± 8.56	11.60 ± 8.22	10.17 ± 8.55	0.23
Subscapularis fold ^b^	13.75 ± 5.66	13.28 ± 5.45	13.67 ± 5.85	0.40
Suprailiac Fold^a^	12.22 ± 4.75	11.45 ± 4.88	12.23 ± 4.73	0.51
FM–Folds (kg) ^a^	14.43 ± 3.91	14.01 ± 3.76	13.96 ± 3.30	0.75

Results are expressed as mean ± standard deviation. A paired *t*-test (^a^) or a nonparametric Wilcoxon test (^b^) was made to assess differences. Statistical significance was assigned as * *p* < 0.05. Abbreviations: Mediterranean Diet (MedDiet); mixed apple and bergamot juice (MAB juice); Standard Deviation (SD); body mass index (BMI); fat-free mass (FFM); total body water (TBW); extracellular water (ECW); body cell mass (BCM); fat mass (FM); phase angle (PA); Sodium (Na); potassium (K); lean mass (LM); metabolic resting rate (BMR); body cell mass index (BCMI).

**Table 4:** Comparison of blood chemistry values between MedDiet and MedDiet + MAB juice groups.

	Baseline *n* = 24 (F = 16)	MedDiet Mean ± SD *n* = 24 (F = 16)	MedDiet + MAB Juice Mean ± SD *n* = 24 (F = 16)	*p* MedDiet vs. MedDiet + MAB Juice	Δ%
TC ^a^	172.45 ± 31.85	169.45 ± 29.65	162.91 ± 25.27	0.20	−3.86
HDL (mg/dL) ^a^	63.82 ± 11.40	63.36 ± 11.62	65.27 ± 10.36	0.39	3.01
LDL (mg/dL) ^a^	92.64 ± 19.89	94.50 ± 19.98	89.00 ± 18.79	0.20	−4.49
TC/HDL ^b^	2.73 ± 0.40	2.74 ± 0.35	2.14 ± 0.73	* <0.01	−20.91
LDL/HDL ^a^	1.48 ± 0.37	1.53 ± 0.35	1.40 ± 0.30	0.08	−7.56
TC/LDL ^a^	1.88 ± 0.21	1.80 ± 0.20	1.84 ± 0.12	0.38	2.22
Glycemia (mg/dL) ^a^	77.83 ± 8.87	77.45 ± 7.94	78.18 ± 8.34	0.73	0.94
Insulin (U/mL) ^a^	5.81 ± 1.57	5.70 ± 1.53	6.23 ± 1.62	0.36	9.30
HOMA-IR ^a^	1.10 ± 0.33	1.08 ± 0.29	1.20 ± 0.32	0.34	11.11

Results are expressed as mean ± standard deviation. A paired *t*-test (^a^) or a nonparametric Wilcoxon test (^b^) was made to assess differences. Statistical significance was assigned as * *p* < 0.05. Abbreviations: Mediterranean Diet (MedDiet); mixed apple and bergamot juice (MAB juice); Standard Deviation (SD); high-density lipoprotein cholesterol (HDL); low-density lipoprotein cholesterol (LDL); total cholesterol (TC); homeostasis model assessment of insulin resistance (HOMA-IR).

## Data Availability

The data presented in this study are available on request from the corresponding author.

## Supplementary Materials

The following supporting information can be downloaded at: https://www.mdpi.com/article/10.3390/genes14010206/s1, Table S1: Determination of nutritional parameters in MAB juice; Table S2. Functional properties of MAB juice.

## References

[1] De Lorenzo A., Cenname G., Marchetti M., Gualtieri P., Dri M., Carrano E., Pivari F., Esposito E., Picchioni O., Moia A. (2022). Social inequalities and nutritional disparities: The link between obesity and COVID-19. Eur. Rev. Med. Pharmacol. Sci..

[2] Ruthsatz M., Candeias V. (2020). Non-communicable disease prevention, nutrition, and aging. Acta Biomed..

[3] Di Renzo L., Gualtieri P., Romano L., Marrone G., Noce A., Pujia A., Perrone M.A., Aiello V., Colica C., De Lorenzo A. (2019). Role of Personalized Nutrition in Chronic-Degenerative Diseases. Nutrients.

[4] Di Renzo L., De Lorenzo A., Fontanari M., Gualtieri P., Monsignore D., Schifano G., Alfano V., Marchetti M., SIERR (2022). Immunonutrients involved in regulating the inflammatory and oxidative processes: Implication for gamete competence. J. Assist. Reprod. Genet..

[5] Soldati L., Di Renzo L., Jirillo E., Ascierto P.A., Marincola F.M., De Lorenzo A. (2018). The influence of diet on anti-cancer immune responsiveness. J. Transl. Med..

[6] Noce A., Marchetti M., Marrone G., Di Renzo L., Di Lauro M., Di Daniele F., Albanese M., Di Daniele N., De Lorenzo A. (2022). Link between gut microbiota dysbiosis and chronic kidney disease. Eur. Rev. Med. Pharmacol. Sci..

[7] De Lorenzo A., Noce A., Bigioni M., Calabrese V., Della Rocca D.G., Di Daniele N., Tozzo C., Di Renzo L. (2010). The effects of Italian Mediterranean organic diet (IMOD) on health status. Curr. Pharm. Des..

[8] De Lorenzo A., Siclari M., Gratteri S., Romano L., Gualtieri P., Marchetti M., Merra G., Colica C. (2019). Developing and cross-valid new equations to estimate fat mass in the Italian population. Eur. Rev. Med. Pharmacol. Sci..

[9] Di Daniele N., Petramala L., Di Renzo L., Sarlo F., Della Rocca D.G., Rizzo M., Fondacaro V., Iacopino L., Pepine C.J., De Lorenzo A. (2013). Body composition changes and cardiometabolic benefits of a balanced Italian Mediterranean Diet in obese patients with metabolic syndrome. Acta Diabetol..

[10] Fabozzi G., Cimadomo D., Allori M., Vaiarelli A., Colamaria S., Argento C., Amendola M.G., Innocenti F., Soscia D., Maggiulli R. (2021). Maternal body mass index associated with blastocyst euploidy and live birth rates: The tip of an iceberg?. Reprod. Biomed. Online.

[11] Di Renzo L., Gualtieri P., Pivari F., Soldati L., Attinà A., Leggeri C., Cinelli G., Tarsitano M.G., Caparello G., Carrano E. (2020). COVID-19: Is there a role for immunonutrition in the obese patient?. J. Transl. Med..

[12] Holt E.M., Steffen L.M., Moran A., Basu S., Steinberger J., Ross J.A., Hong C.P., Sinaiko A.R. (2009). Fruit and vegetable consumption and its relation to markers of inflammation and oxidative stress in adolescents. J. Am. Diet. Assoc..

[13] Di Renzo L., Gualtieri P., Alwardat N., De Santis G., Zomparelli S., Romano L., Marchetti M., Michelin S., Capacci A., Piccioni A. (2020). The role of IL-6 gene polymorphisms in the risk of lipedema. Eur. Rev. Med. Pharmacol. Sci..

[14] Zou H., Ye H., Kamaraj R., Zhang T., Zhang J., Pavek P. (2021). A review on pharmacological activities and synergistic effect of quercetin with small molecule agents. Phytomedicine.

[15] Leontowicz M., Gorinstein S., Leontowicz H., Krezeminski R., Lojek A., Katrich E., Ciz M., Martin B. (2003). Apple and pear peel and pulp and their influences on plasma lipids and antioxidant potentialin rats fed cholesterol-containing diets. J. Agric. Food Chem..

[16] Grobelna A., Kalisz S., Kieliszek M. (2019). The Effect of the Addition of Blue Honeysuckle Berry Juice to Apple Juice on the Selected Quality Characteristics, Anthocyanin Stability, and Antioxidant Properties. Biomolecules.

[17] Aprikian O., Duclos V., Guyot S., Besson C., Manach C., Bernalier A., Morand C., Rémésy C., Demigné C. (2003). Apple pectin and a polyphenol-rich apple concentrate are more effective together than separately on cecal fermentations and plasma lipids in rats. J. Nutr..

[18] Kamdi S.P., Raval A., Nakhate K.T. (2021). Effect of apple peel extract on diabetes-induced peripheral neuropathy and wound injury. J. Diabetes Metab. Disord..

[19] De Oliviera M., Sichieri R., Moura A. (2003). Weight loss associated with a daily intake of three apples or three pears among over weight women. Nutrition.

[20] Gattuso G., Caristi C., Gargiulli C., Bellocco E., Toscano G., Leuzzi U. (2011). Flavonoid Glycosides in Bergamot Juice (Citrus bergamia Risso). J. Agric. Food Chem..

[21] Sindona G., Di Donna L., Dolce V. (2010). WO 2010/041290 A1, Natural Molecule Extracted from Bergamot Tissues, Extraction Process and Pharmaceutical Use.

[22] Da Pozzo E., De Leo M., Faraone I., Milella L., Cavallini C., Piragine E., Testai L., Calderone V., Pistelli L., Braca A. (2018). Antioxidant and Antisenescence Effects of Bergamot Juice. Oxid. Med. Cell. Longev..

[23] Miceli N., Mondello M.R., Monforte M.T., Sdrafkakis V., Dugo P., Crupi M.L., Taviano M.F., De Pasquale R., Trovato A. (2007). Hypolipidemic effects of Citrus bergamia Risso et Poiteau juice in rats fed a hypercholesterolemic diet. J. Agric. Food Chem..

[24] Mollace V., Sacco I., Janda E., Malara C., Ventrice D., Colica C., Visalli V., Muscoli S., Ragusa S., Muscoli C. (2011). Hypolipemic and hypoglycaemic activity of bergamot polyphenols: From animal models to human studies. Fitoterapia.

[25] Leopoldini M., Malaj N., Toscano M., Sindona G., Russo N. (2010). On the inhibitor effects of bergamot juice flavonoids binding to the 3-hydroxy-3-methylglutaryl-CoA reductase (HMGR) enzyme. J. Agric. Food Chem..

[26] Watson R.R., Victor R., Zibadi P.S. (2013). Polyphenols in Human Health and Disease, 1st ed.

[27] Colica C., Di Renzo L., Trombetta D., Smeriglio A., Bernardini S., Cioccoloni G., Costa de Miranda R., Gualtieri P., Sinibaldi Salimei P., De Lorenzo A. (2017). Antioxidant Effects of a Hydroxytyrosol-Based Pharmaceutical Formulation on Body Composition, Metabolic State, and Gene Expression: A Randomized Double-Blinded, Placebo-Controlled Crossover Trial. Oxid. Med. Cell. Longev..

[28] Di Renzo L., Merra G., Botta R., Gualtieri P., Manzo A., Perrone M.A., Mazza M., Cascapera S., De Lorenzo A. (2017). Post-prandial effects of hazelnut-enriched high fat meal on LDL oxidative status, oxidative and inflammatory gene expression of healthy subjects: A randomized trial. Eur. Rev. Med. Pharmacol. Sci..

[29] Rapporti ISTISAN 1996/34. https://www.iss.it/documents/20126/45616/Rapp_ISTISAN_96_34_def.pdf.

[30] Smeriglio A., Denaro M., D’Angelo V., Germanò M.P., Trombetta D. (2020). Antioxidant, Anti-Inflammatory and Anti-Angiogenic Properties of Citrus lumia Juice. Front. Pharmacol..

[31] Merra G., Gualtieri P., Cioccoloni G., Falco S., Bigioni G., Tarsitano M.G., Capacci A., Piccioni A., Costacurta M., Franceschi F. (2020). FTO rs9939609 influence on adipose tissue localization in the Italian population. Eur. Rev. Med. Pharmacol. Sci..

[32] Merra G., Miranda R., Barrucco S., Gualtieri P., Mazza M., Moriconi E., Marchetti M., Chang T.F., De Lorenzo A., Di Renzo L. (2016). Very-low-calorie ketogenic diet with aminoacid supplement versus very low restricted-calorie diet for preserving muscle mass during weight loss: A pilot double-blind study. Eur. Rev. Med. Pharmacol. Sci..

[33] Di Renzo L., Cinelli G., Romano L., Zomparelli S., Lou De Santis G., Nocerino P., Bigioni G., Arsini L., Cenname G., Pujia A. (2021). Potential Effects of a Modified Mediterranean Diet on Body Composition in Lipoedema. Nutrients.

[34] Di Renzo L., Marchetti M., Cioccoloni G., Gratteri S., Capria G., Romano L., Soldati L., Mele M.C., Merra G., Cintoni M. (2019). Role of phase angle in the evaluation of effect of an immuno-enhanced formula in post-surgical cancer patiens: A randomized clinical trial. Eur. Rev. Med. Pharmacol. Sci..

[35] Romano L., Marchetti M., Gualtieri P., Di Renzo L., Belcastro M., De Santis G.L., Perrone M.A., De Lorenzo A. (2019). Effects of a Personalized VLCKD on Body Composition and Resting Energy Expenditure in the Reversal of Diabetes to Prevent Complications. Nutrients.

[36] Di Renzo L., Gratteri S., Sarlo F., Cabibbo A., Colica C., De Lorenzo A. (2014). Individually tailored screening of susceptibility to sarcopenia using p53 codon 72 polymorphism, phenotypes, and conventional risk factors. Dis. Markers.

[37] Di Renzo L., Galvano F., Orlandi C., Bianchi A., Di Giacomo C., La Fauci L., Acquaviva R., De Lorenzo A. (2010). Oxidative stress in normal-weight obese syndrome. Obesity.

[38] Di Renzo L., Cioccoloni G., Sinibaldi Salimei P., Ceravolo I., De Lorenzo A., Gratteri S. (2018). Alcoholic Beverage and Meal Choices for the Prevention of Noncommunicable Diseases: A Randomized Nutrigenomic Trial. Oxid. Med. Cell. Longev..

[39] De Lorenzo A., Romano L., Di Renzo L., Di Lorenzo N., Cenname G., Gualtieri P. (2020). Obesity: A preventable, treatable, but relapsing disease. Nutrition.

[40] Fitó M., Konstantinidou V. (2016). Nutritional Genomics and the Mediterranean Diet’s Effects on Human Cardiovascular Health. Nutrients.

[41] Gkouskou K., Lazou E., Skoufas E., Eliopoulos A.G. (2021). Genetically Guided Mediterranean Diet for the Personalized Nutritional Management of Type 2 Diabetes Mellitus. Nutrients.

[42] Di Renzo L., Gualtieri P., De Lorenzo A., Capacci A., Merra G. (2020). The effective cost of healthy diet. Eur. Rev. Med. Pharmacol. Sci..

[43] Merra G., Noce A., Marrone G., Cintoni M., Tarsitano M.G., Capacci A., De Lorenzo A. (2020). Influence of Mediterranean Diet on Human Gut Microbiota. Nutrients..

[44] Byrd-Bredbenner C., Ferruzzi M.G., Fulgoni V.L., Murray R., Pivonka E., Wallace T.C. (2017). Satisfying America’s fruit gap: Summary of an expert roundtable on the role of 100% fruit juice. J. Food Sci..

[45] Agarwal S., Fulgoni V.L., Welland D. (2019). Intake of 100% fruit juice is associated with improved diet quality of adults: NHANES 2013–2016 analysis. Nutrients.

[46] Zhao S., Bomser J., Joseph L., DiSilvestro R.A. (2013). Intakes of apples or apple polyphenols decease plasma values for oxidized low-density lipoprotein/beta2-glycoprotein I complex. J. Funct. Foods.

[47] da Silva Porto P.A., Laranjinha J.A., de Freitas V.A. (2003). Antioxidant protection of low density lipoprotein by procyanidins: Structure/activity relationships. Biochem. Pharmacol..

[48] Vallée Marcotte B., Verheyde M., Pomerleau S., Doyen A., Couillard C. (2022). Health Benefits of Apple Juice Consumption: A Review of Interventional Trials on Humans. Nutrients.

[49] Boyer J., Liu R.H. (2004). Apple phytochemicals and their health benefits. Nutr. J..

[50] Kschonsek J., Wolfram T., Stöckl A., Böhm V. (2018). Polyphenolic compounds analysis of old and new apple cultivars and contribution of polyphenolic profile to the in vitro antioxidant capacity. Antioxidants.

[51] Clemens R., Drewnowski A., Ferruzzi M.G., Toner C.D., Welland D. (2015). Squeezing fact from fiction about 100% fruit juice. Adv. Nutr. Int. Rev. J..

[52] Wruss J., Lanzerstorfer P., Huemer S., Himmelsbach M., Mangge H., Höglinger O., Weghuber D., Weghuber J. (2015). Differences in pharmacokinetics of apple polyphenols after standardized oral consumption of unprocessed apple juice. Nutr. J..

[53] Barth S.W., Koch T.C.L., Watzl B., Dietrich H., Will F., Bub A. (2012). Moderate effects of apple juice consumption on obesity-related markers in obese men: Impact of diet–gene interaction on body fat content. Eur. J. Nutr..

[54] Ravn-Haren G., Dragsted L.O., Buch-Andersen T., Jensen E.N., Jensen R.I., Németh-Balogh M., Paulovicsová B., Bergström A., Wilcks A., Licht T.R. (2013). Intake of whole apples or clear apple juice has contrasting effects on plasma lipids in healthy volunteers. Eur. J. Nutr..

[55] White S.J., Carran E.L., Reynolds A.N., Haszard J.J., Venn B.J. (2018). The effects of apples and apple juice on acute plasma uric acid concentration: A randomized controlled trial. Am. J. Clin. Nutr..

[56] Vieira F.G., Di Pietro P.F., Da Silva E.L., Borges G., Nunes E.C., Fett R. (2012). Improvement of serum antioxidant status in humans after the acute intake of apple juices. Nutr. Res..

[57] Mandalari G., Bennett R.N., Bisignano G., Trombetta D., Saija A., Faulds C.B., Gasson M.J., Narbad A. (2007). Antimicrobial activity of flavonoids extracted from bergamot (Citrus bergamia Risso) peel, a byproduct of the essential oil industry. J. Appl. Microbiol..

[58] Hyson D., Studebaker-Hallman D., Davis P.A., Gershwin M.E. (2000). Apple juice consumption reduces plasma low-density lipoprotein oxidation in healthy men and women. J. Med. Food.

[59] Soriano-Maldonado A., Hidalgo M., Arteaga P., de Pascual-Teresa S., Nova E. (2014). Effects of regular consumption of vitamin C-richor polyphenol-rich apple juice on cardiometabolic markers in healthy adults: A randomized crossover trial. Eur. J. Nutr..

[60] Wilcox L.J., Borradaile N.M., de Dreu L.E., Huff M.W. (2001). Secretion of hepatocyte apoB is inhibited by the flavonoids, naringenin and hesperetin, via reduced activity and expression of ACAT2 and MTP. J. Lipid Res..

[61] Caradonna F., Consiglio O., Luparello C., Gentile C. (2020). Science and Healthy Meals in the World: Nutritional Epigenomics and Nutrigenetics of the Mediterranean Diet. Nutrients.

[62] Konstantinidou V., Covas M.I., Sola R., Fitó M. (2013). Up-to date knowledge on the in vivo transcriptomic effect of the Mediterranean diet in humans. Mol. Nutr. Food Res..

[63] Jung M., Triebel S., Anke T., Richling E., Erkel G. (2009). Influence of apple polyphenols on inflammatory gene expression. Mol. Nutr. Food Res..

[64] Xie J., Zhang X., Zhang L. (2013). Negative regulation of inflammation by SIRT1. Pharmacol. Res..

[65] Borgatti M., Mancini I., Bianchi N., Guerrini A., Lampronti I., Rossi D., Sacchetti G., Gambari R. (2011). Bergamot (Citrus bergamia Risso) fruit extracts and identified components alter expression of interleukin 8 gene in cystic fibrosis bronchial epithelial cell lines. BMC Biochem..

[66] Risitano R., Currò M., Cirmi S., Ferlazzo N., Campiglia P., Caccamo D., Ientile R., Navarra M. (2014). Flavonoid fraction of Bergamot juice reduces LPS-induced inflammatory response through SIRT1-mediated NF-κB inhibition in THP-1 monocytes. PLoS ONE.

[67] Impellizzeri D., Bruschetta G., Di Paola R., Ahmad A., Campolo M., Cuzzocrea S., Navarra M. (2015). The anti-inflammatory and antioxidant effects of bergamot juice extract (BJe) in an experimental model of inflammatory bowel disease. Clin. Nutr..

[68] Werner T. (2001). Cluster analysis and promoter modelling as bioinformatics tools for the identification of target genes from expression array data. Pharmacogenomics.

[69] Di Renzo L., Cioccoloni G., Bernardini S., Abenavoli L., Aiello V., Marchetti M., Cammarano A., Alipourfard I., Ceravolo I., Gratteri S. (2019). A Hazelnut-Enriched Diet Modulates Oxidative Stress and Inflammation Gene Expression without Weight Gain. Oxid. Med. Cell. Longev..

[70] Osipyan A., Chen D., Dekker F.J. (2021). Epigenetic regulation in macrophage migration inhibitory factor (MIF)-mediated signaling in cancer and inflammation. Drug Discov. Today.

[71] Cooke G., Armstrong M.E., Donnelly S.C. (2009). Macrophage migration inhibitory factor (MIF), enzymatic activity and the inflammatory response. Biofactors.

[72] Rius-Pérez S., Pérez S., Martí-Andrés P., Monsalve M., Sastre J. (2020). Nuclear Factor Kappa B Signaling Complexes in Acute Inflammation. Antioxid. Redox Signal..

[73] Hernandez-Quiles M., Broekema M.F., Kalkhoven E. (2021). PPARgamma in Metabolism, Immunity, and Cancer: Unified and Diverse Mechanisms of Action. Front. Endocrinol..

[74] Kliewer S.A., Sundseth S.S., Jones S.A., Brown P.J., Wisely G.B., Koble C.S., Devchand P., Wahli W., Willson T.M., Lenhard J.M. (1997). Fatty acids and eicosanoids regulate gene expression through direct interactions with peroxisome proliferator-activated receptors alpha and gamma. Proc. Natl. Acad. Sci. USA.

[75] Wang Y., Zhu J., DeLuca H.F. (2012). Where is the vitamin D receptor?. Arch. Biochem. Biophys..

[76] Pike J.W., Meyer M.B., Bishop K.A. (2012). Regulation of target gene expression by the vitamin D receptor—An update on mechanisms. Rev. Endocr. Metab. Disord..

[77] Castrop H. (2015). A role for AT1 receptor-associated proteins in blood pressure regulation. Curr. Opin. Pharmacol..

[78] Al-Wardat M., Alwardat N., Lou De Santis G., Zomparelli S., Gualtieri P., Bigioni G., Romano L., Di Renzo L. (2021). The association between serum vitamin D and mood disorders in a cohort of lipedema patients. Horm. Mol. Biol. Clin. Investig..

[79] Lei M., Liu Z., Guo J. (2020). The Emerging Role of Vitamin D and Vitamin D Receptor in Diabetic Nephropathy. Biomed. Res. Int..

[80] Papandreou D., Magriplis E., Abboud M., Taha Z., Karavolia E., Karavolias C., Zampelas A. (2019). Consumption of Raw Orange, 100% Fresh Orange Juice, and Nectar- Sweetened Orange Juice-Effects on Blood Glucose and Insulin Levels on Healthy Subjects. Nutrients.

[81] Tsilas C.S., de Souza R.J., Mejia S.B., Mirrahimi A., Cozma A.I., Jayalath V.H., Ha V., Tawfik R., Di Buono M., Jenkins A.L. (2017). Relation of total sugars, fructose and sucrose with incident type 2 diabetes: A systematic review and meta-analysis of prospective cohort studies. CMAJ.

