# Supplementary material for: Antioxidant-Enriched Diet on Oxidative Stress and Inflammation Gene Expression: A Randomized Controlled Trial
Source: Genes (Basel). 2023 Jan 13;14(1):206. doi: 10.3390/genes14010206 (PMC9859217; doi:10.3390/genes14010206)
Supplement: Supplementary file 1 [file genes-14-00206-s001.zip › genes-2100008-supplementary.pdf]

Table S1. Determination of nutritional parameters in MAB juice.

Nutrients (g/100 g)	MAB juice
Energy value (kcal/kJ)	35/149
Humidity	91.03 ± 1.27
Ash	0.23 ± 0.01
Sodium	<0.02 ± 0.00
Salt	0.002 ± 0.00
Protein	0.19 ± 0.01
Fats	<0.1 ± 0.00
Monounsaturated fatty acids	<0.1 ± 0.00
Polyunsaturated fatty acids	<0.1 ± 0.00
Saturated fatty acids	<0.1 ± 0.00
Carbohydrates of which:	8.55 ± 0.18
Sugars	8.55 ± 0.18
Dietary fiber	<0.5 ± 0.01

Data, which represent the average ± standard deviations (SD) of three independent experiments (n =3), were expressed as g/100 g of juice. Abbreviations: standard deviations (SD); mixed apple and bergamot juice (MAB juice).

Table S2. Functional properties of MAB juice.

Compound	RT (min)	λ_{max} (nm)	$[M\text{-}H]^-$	mg/L
Procyanidin B1	18.48	280	577	4.40 ± 0.08
**Chlorogenic acid**	20.29	326	353	30.08 ± 0.25
(+)-Catechin	20.56	278	289	3.04 ± 0.05
**Procyanidin B2**	21.71	280	577	30.96 ± 0.37
Vicenin-2	20.76	335	593	7.72 ± 0.12
Caffeic acid	21.95	325	179	3.84 ± 0.04
**(–)-Epicatechin**	22.41	278	289	37.28 ± 0.18
**4-p-Cumaroylquinic acid**	22.87	312	337	11.52 ± 0.15
Stellarin-2	23.94	347	623	5.16 ± 0.05
Quercetin-3-*O*-rhamnoside	29.57	350	447	1.28 ± 0.02
Phloretin-2'-*O*-xyloglucoside	30.03	284	567	6.08 ± 0.04
Phloridzin	32.11	284	435	3.28 ± 0.02
**Neoeriocitrin**	32.89	285	595	14.66 ± 0.22
**Naringin**	39.08	285	579	33.50 ± 0.33
Rhoifolin	39.64	340	577	9.28 ± 0.06
Neodiosmin	42.08	350	607	4.62 ± 0.02
Neoeriocitrin-di-oxalate	42.26	284	739	4.02 ± 0.04
**Neohesperidin**	42.82	285	609	24.78 ± 0.38
**Meltidin**	46.87	285	707	85.24 ± 0.67
**Naringin-di-oxalate**	47.69	284	723	17.86 ± 0.16
**Brutieridin**	47.92	285	737	108.27 ± 1.44
**Neohesperidin-di-oxalate**	50.86	284	753	38.00 ± 0.52
Vitamin C (mg/L)				422.02 ± 1.88
TPC (mg GAE/L)				1263.16 ± 8.53
TFC (mg QE/L)				385.38 ± 2.66
ORAC (μmoli TE/L)				5964.29 ± 12.48

Results are expressed as mean ± standard deviation of three independent experiments in triplicate (n =3) for each parameter. Polyphenolic profile characterization and Vitamin C content were established by LC-DAD-ESI-MS and HPLC-DAD analysis, respectively. TPC, TFC, and ORAC value were quantified by spectrophotometric and spectrofluorimetric assays, respectively. The most abundant polyphenols were reported in bold. Abbreviations: total phenolic compound (TPC); gallic acid equivalents (GAE); total flavonoid compound (TFC); quercetin equivalents (QE); Oxygen radical absorbance capacity (ORAC); Trolox equivalents (TE).
